# Age differences in bonobo (*Pan paniscus*) multimodal communication signals

**DOI:** 10.1007/s10071-025-01961-2

**Published:** 2025-05-19

**Authors:** Elizabeth Beachem, Caleb Ghione, Halena Soto, Lisette van den Berg, Craig Stanford

**Affiliations:** 1 Department of Integrative and Evolutionary Biology, University of Southern California, Los Angeles, CA USA; 2 Department of Molecular and Computational Biology, University of Southern California, Los Angeles, CA USA; 3 Department of Anthropology, University of California, Santa Cruz, Santa Cruz, CA USA; 4 Apenheul Primate Park, Apeldoorn, the Netherlands

**Keywords:** Bonobo, Multimodal signals, Primate communication, Language evolution

## Abstract

While spoken language is unique to humans, many features of human communication are shared with great apes, including the use of signals in multiple modalities such as vocalizations, gestures, and facial expressions. Communication signals can be unimodal (involving a single modality) or multimodal (combining multiple modalities simultaneously). Here, we examined age-related differences in bonobo (*Pan paniscus)* unimodal and multimodal communication signals. We assessed all vocalizations, gestures, facial expressions, and multimodal combinations produced by captive bonobos across a variety of behavioral contexts. All occurrences of communication signals were collected via focal observations from 12 individuals ranging from 6 months to 44 years of age. All individuals produced multimodal communication signals but all bonobos, regardless of age, produced multimodal signals at lower frequencies than unimodal signals. Age had a significant effect with younger bonobos producing more multimodal signals than older individuals (*p* < 0.001). The infant and juveniles produced the most multimodal signals and there was an approximately 6% increase in unimodal signals per age year increase. These findings indicate a developmental shift toward unimodal signals as bonobos age. Behavioral context was predictive of signal type usage with an increase of multimodal signals in agonistic (*p* < 0.001), play (*p* < 0.001), and sexual contexts (*p* = 0.001). This indicates that context is important for bonobo modality with multimodal signaling occurring more in “high-risk/high-reward” contexts where proper signal comprehension is vital. This study represents an overview of multimodal communication across bonobo life stages, offering further insights into primate communication patterns and developmental trajectories.

**Supplementary Information:**

The online version contains supplementary material available at 10.1007/s10071-025-01961-2.

## Introduction

Language is a mixed-media communication system featuring various types of signals in different modalities such as facial expressions, vocalization, and gestures. While humans are thought to be unique in having language, it is likely that some or all components were present in the last common ancestor of humans, chimpanzees (*Pan troglodytes*), and bonobos (*Pan paniscus*) (Pollick and de Waal 2007; Arbib et al. 2008; Birchenall 2016; Byrne et al. 2017). As language does not fossilize, we look at the behaviors of genetically similar primates to seek shared ancestral traits or homologies (Fedurek and Slocombe 2011; Rodrigues et al. 2021). Members of the genus *Pan*, bonobos and chimpanzees, are our closest living ancestors, sharing over 98% of our human DNA (Luke and Verma 1995; Prüfer et al. 2012), and are frequently used as models for extinct early humans (Jensen-Seaman et al. 2001; Sayers and Lovejoy 2008; D’Août et al. 2014). Investigating shared communication patterns in humans and great apes (hereafter, apes) allows us to better understand the fundamental building blocks of language (Fisher and Marcus 2006; Arbib et al. 2008; Taglialatela et al. 2008).

Communication signals can be unimodal or multimodal: unimodal signals are communicative signals sent through one modality (e.g. a gesture or a vocalization), whereas multimodal signals are signals in more than one modality produced at the same time (e.g. a gesture and vocalization combined) (Partan and Marler 1999, 2005; Higham and Hebets 2013). In this study, we consider multimodal signals as overlapping signals produced in more than one modality (vocal/gestural/facial) that occur simultaneously with one another (Wilke et al. 2017; Fröhlich et al. 2019; Genty 2019). Researchers argue that it may be the combination of unimodal signals into multimodal signals that led to the evolution of spoken language, making multimodal signaling a key trait of language (Slocombe et al. 2011; Taglialatela et al. 2011; Gillespie-Lynch et al. 2014; Levinson and Holler 2014; Vigliocco et al. 2014; Fröhlich et al. 2019). While language likely has deep evolutionary roots shared among living apes, most studies have used a unimodal research approach (e.g., vocalizations: Seyfarth et al. 1994; Fedurek and Slocombe 2011; gestures: Pika et al. 2005; Cartmill and Byrne 2010; facial expressions: Waller et al. 2015). When we consider the wide array of gestures primates use in addition to vocalizations and facial expressions, the potential multimodal combinations previously neglected are abundant. Within the genus *Pan*, bonobos rather than chimpanzees, have been shown to respond more to multimodal signals than most unimodal signals (Pollick and de Waal 2007). Our study takes this knowledge and focuses on bonobos as a potentially more fruitful model for studying the production and usage of multimodal signals.

When studying multimodal signals in apes, it is important to distinguish between multimodal signals that apes are free to combine and those that are obligatorily combined. To be considered a true multimodal signal, both components of differing modalities cannot be mechanically tied and always occur together (Partan and Marler 2005; Higham and Hebets 2013). For instance, in order to produce a “pant-hoot” vocalization, chimpanzees must press and purse their lips, as the facial expression is required to produce the sound. Free multimodal signals, instead, occur when an individual can flexibly and optionally produce the different modality components, like a human waving “hello” while vocalizing the word (Wilke et al. 2017; Fröhlich and Hobaiter 2018). All multimodal communication signals captured in this study are free signals and were not produced as a necessary function of their signal (See example of a multimodal signal combination in Supplementary Fig. S1).

Multimodal communication research in the apes has been done in both captive and wild populations but remains relatively understudied compared to unimodal signals (Leavens et al. 2004, 2009; Parr 2004; Forrester and Forrester 2005; Leavens 2007; Pollick and de Waal 2007; Slocombe et al. 2011; Genty et al. 2014; Taglialatela et al. 2015; Hobaiter et al. 2017; Wilke et al. 2017; Genty 2019; Doherty et al. 2023). While research has, to some degree, studied multimodal signaling, the current literature on ape multimodal communication has rarely looked at how the developmental trajectory across ontogeny affects multimodal communication (Bard et al. 2014; Gillespie-Lynch et al. 2014; Fröhlich et al. 2016; Dafreville et al. 2021; Doherty et al. 2023). Studies have shown that individuals in different life stages have unique ways of communicating as they go through the process of forming their communicative repertoire (Schneider et al. 2011; Bard et al. 2014). In bonobos, while most gestures are produced and understood by all individuals, there are some gestures that are only part of older individuals’ repertoire (Graham et al. 2016). This indicates that certain communication signals are produced and understood by specific age categories and could reflect the different developmental needs that individuals face as they age. Early life stages in humans are crucial periods for learning communication and language (Mayberry et al. 2002; Tsao et al. 2004; Kuhl 2010). In nonhuman primates, the younger life stages are also important, and individuals may refine their communication skills by learning from other group members through social experiences (Flack et al. 2004; Hobaiter and Byrne 2011b; Weisberg et al. 2013; Palagi et al. 2015, Fröhlich et al. 2018; Amici and Liebal 2022).

In captive chimpanzees, infants start using multimodal signals at 18 weeks of age (Bard et al. 2014). It has been proposed that younger individuals produce rapid sequences of signals more frequently because they have not yet learned their meanings; a process the coining authors refer to as the “repertoire tuning hypothesis” (Hobaiter and Byrne 2011a). The same mechanism could also underlie the production of multimodal signals: multimodal combinations decrease through age, when older apes can use a set of defined unimodal signals, as they have gradually learned the meaning of specific signals or can use signals more effectively and thus rely more on unimodal signals later in life. Recently, researchers found support for a patterns of age and multimodal signaling in semi-wild chimpanzees, with early adolescents producing more multimodal combinations than infants and juveniles (Doherty et al. 2023). In bonobos, individuals over 2 years of age displayed more functional specificity in their multimodal signals than younger bonobos (Genty 2019). Although these studies show that age has some impact on signal modality in chimpanzees and bonobos, there are, to our knowledge, no known comprehensive studies that capture all modalities (vocalizations, gestures, facial expressions, and multimodal combinations) while comparing multiple age categories in bonobos.

Primates living in large social groups have to navigate social relationships across a variety of contexts in which communication with other individuals is an important part of everyday life. A function of multimodal communication signals is to aid in proper receiver comprehension by combining modalities (Genty et al. 2014; Hobaiter et al. 2017; Wilke et al. 2017; Fröhlich et al. 2019; Oña et al. 2019). In behavioral contexts that have high risk or reward, such as aggression or sexual encounters, signal comprehension is vital. Hobaiter et al. (2017) found that there were higher levels of multimodal signals sent during agonistic contexts in wild chimpanzees. Doherty et al. (2023) found a similar effect in a chimpanzee sanctuary setting; multimodal combinations were seen most often in agonistic contexts by adolescents. Bonobos are known for their use of sociosexual behaviors to resolve conflict and reinforce social bonds, and the use of multimodal signals during these behaviors may help this process (de Waal 1990; Wrangham 1993; Hashimoto 1997; Manson et al. 1997; Parish et al. 2000). In sexual contexts, researchers have found that bonobo multimodal signals are produced more during initiations (Genty et al. 2014). In addition to these contexts, communication studies in apes often look at signals sent during play and have found it to be one of the most prolific communicative contexts (Tanner and Perlman 2017; Oliveira and Waisterlain 2020). We know that primates employ specific facial expressions and vocalizations during play to ensure that rougher gestures are not received aggressively by their partners (Chevalier-Skolnikoff 1974; Waller and Dunbar 2005; Tomasello 2008; Palagi et al. 2015). Genty et al. (2014) studied contest hoot vocalizations in bonobos and found that while they are used unimodally in agonistic events, bonobos combine them multimodally with affiliative gestures during play contexts. Doherty et al. (2023) found that chimpanzees from infancy through adolescence showed higher proportions of multimodal signals in play contexts regardless of age. In this study, we assessed communication signals with the hypothesis that behavioral contexts would affect the rates of multimodal signals. We predicted that the proportion of multimodal signals would increase in “high-risk/high-reward” contexts such as agonism and sexual interactions (Prediction 1a). In these contexts, individuals might need to use combined modalities to convey their meaning clearly to their receivers. Additionally, we expected to see increased rates of multimodality during play contexts following the trends displayed in the literature (Prediction 1b).

The present study addresses the multimodal communication knowledge gap by investigating and comparing unimodal and multimodal signals in bonobos across ontogeny. We employed a comprehensive all-occurrence sampling model to capture any communicative signals- vocal, gestural, facial, and multimodal signals in a variety of behavioral contexts. We predicted that, in line with the repertoire tuning hypothesis for sequential signals (Hobaiter and Byrne 2011a), bonobos would display similar developmental trajectories as chimpanzees, with multimodal signals being more likely produced by younger than older bonobos (Prediction 2).

## Methods

### Study site and subjects

This study took place at Apenheul Primate Park, hereafter Apenheul, in Apeldoorn, the Netherlands. Apenheul houses a colony of 12 bonobos of both sexes across multiple age categories (M = 5, F = 7). Bonobos are kept in two interchangeable subgroups ranging from 2 to 8 individuals that are changed at least once a week to mimic the fission-fusion lifestyle of wild bonobos. Apenheul has a combination of indoor and outdoor enclosures. Indoor enclosures are composed of 3 rooms viewable by zoo visitors and 3 enclosures below the ground floor of the building (total ~ 200m^2^). The outdoor island, available to the bonobos when the temperature is above 5℃C, is approximately 4,600 m² and is surrounded by a 1.5 m deep moat. There are trees, natural vegetation, and water streams across the island as well as constructed climbing structures and a termite mound. Bonobos are fed a diet consisting mainly of vegetables, browse, nuts, grains, and vitamin-enriched chow biscuits. Food is provided four to five times a day using various techniques and enrichment devices, and water is available *ad libitum*.

The bonobo colony consisted of individuals from infancy to adulthood. Based on previous studies, age classifications were adapted (de Waal 1988; Palagi 2006; Schneider et al. 2017). We considered infants all individuals under the age of 3 years, juveniles as bonobos between 3 and 6 years, adolescents between 7 and 12 years of age, and individuals over the age of 12 as adults (Table 1).

**Table 1:** Descriptive information on observed bonobos (*n =* 12)

Age category	Age (year)	Individual & Sex	Observation time (hours)
Infant	0.5	Neje (F)	41.25
Juvenile	3	Eyenga (M)	40.25
	3	Lokolo (M)	39.50
Adolescent	7	Ayebi (F)	31.50
	12	Makasi (M)	27.00
	12	Pangi (F)	28.50
Adult	15	Besede (F)	30.50
	23	Bolombo (M)	29.00
	44	Bonnie (F)	24.25
	36	Jill (F)	26.25
	37	Kindu (M)	23.00
	22	Kumbuka (F)	30.75

### Data collection

Live observational data and video recordings were collected by the first author during September-November 2021 and May-December 2022, using focal animal sampling (Altmann 1974). Focal individuals were observed for 15 minutes. Observations were quasi-randomized and taken opportunistically between 9 am and 5 pm. If a focal animal was out of sight for more than half of the observation length, the observation was terminated and discarded. Observations were recorded with a Canon EOS Rebel DSLR camera with a mounted Rode VideoMic Pro + Directional Shotgun Microphone. We preferentially recorded bonobos outdoors, but when this was not possible, we observed them indoors, recording sound with a battery-operated wireless camera (Wyze Outdoor V2).

We recorded all occurrences of communicative signals from the focal individual in real-time using an Apple 2019 iPad Pro and the ZooMonitor app (Ross et al. 2016). Signals were classified based on an ethogram including vocalizations, facial expressions, and gestures that we created based on previous literature and from the Great Ape Dictionary (de Waal 1988; Bermejo and Omedes 1999; Pika et al. 2005; Pollick and de Waal 2007; Genty et al. 2014; Graham et al. 2016). The ethogram included 10 vocalizations, 4 common facial expressions, 33 gestures, 4 types of multimodal signal combinations, as well as an “other” category for undefined signals in each modality (Supplementary Tables S1-S3).

### Behavioral coding

Observations were coded in real-time as noted above and any videos containing communication signals were identified for further analyses post-data collection using the Behavioral Observation Research Interactive Software (BORIS) (*n* = 1,183 focal observations). The mean observation time across these videos was 30.98 ± 6.19 h per individual. For every communicative signal on the videos, we noted: (*a)* whether it was a unimodal signal or a multimodal combination, (*b*) signal type(s): vocalization, gesture, facial expression, or multimodal combination, (*c*) signal sender, (*d*) sender’s age, (*e*) sender’s sex, (*f*) apparent receiver, (*g*) receiver’s age (*h*) receiver’s sex, and (*i*) behavioral context. Multimodal signal combinations (*b)* were noted as vocalization + gestures (VOC + GES), gestures + facial expressions (GES + FAC), facial expressions + vocalization (FAC + VOC), and as VOC + FAC + GES for a combination of all 3 modalities (vocalization + facial expression + gesture). Signals coded in all modalities are noted in Table 2. Behavioral contexts for signals (*i)* were: affiliative, agonistic, feeding, grooming, locomotion, play, rest, sexual, and “other” (Pollick and de Waal 2007; Hobaiter et al. 2017; Wilke et al. 2017; Supplementary Table S4). The “other” context included things such as defecation/urination, caretaker interactions, coprophagy, etc. Files were coded in BORIS program version 8.21.1-8 with a Windows 11 Dell Inspiron laptop (Friard and Gamba 2016).

**Table 2:** Signals coded for the communication modalities

Modality	Number	Signal
Facial expression	5	Grimace, kiss face, other facial, play face, raspberry face
Gesture	34	Arm raise, bang, bite, bow, clap, drag/pull, embrace, foot raise, genital inspect, genital present, grab, head shake, headbutt, hit, kick, open palm, other gesture, pat, pirouette, play jump, point, poke, pounce, push, rap knuckles, reach, self-hit, somersault, stomp, swagger, swat, sway, throw, touch
Vocalization	11	Alarm call, bark, copulation squeal, grunt, hoot, laugh, other vocalization, peep, pout-moan, raspberry blow, scream
Multimodal combination	4	Facial expression + vocalization (FAC + VOC), gesture + facial expression (GES + FAC), vocalization + gesture (VOC + GES), vocalization + facial expression + gesture (VOC + FAC + GES)

The majority of videos were coded by the first author and approximately 24% were coded by the third author, who was trained in the video coding protocol. Intercoder reliability was tested across 6% of the videos from the dataset (i.e. 70 focal recordings), distributed equally across all 12 sample individuals. Cohen’s kappa was calculated using the “kappa2” function within the IRR package in R (Cohen 1960; Gamer et al. 2022). Mean kappa values were calculated for each modality and multimodal signals with high levels of agreement suggesting excellent consistency between coders (facial expressions = 0.85, gestures = 0.87, vocalizations = 0.81, multimodal = 0.84).

### Statistical analyses

Statistical analyses were run in R (version 4.3.1) and RStudio (2023.09.1 + 494) on a MacOS system (R Core Team 2023). We ran two different full models to test whether the production of multimodal versus unimodal signals varied as a function of behavioral context (Prediction 1) and bonobo signal sender’s age (Prediction 2). To assess this, we ran generalized linear mixed models (GLMMs) using the “glmmTMB” function within the “glmmTMB” R package (Brooks et al. 2017). A total of 1,183 focal observations containing 7,617 communication signals were used for analysis. In the first full model, our binomial response was whether the signal was unimodal or multimodal (0 = unimodal, 1 = multimodal). We included signal sender age in years as a continuous predictor (ranging from 0.5 to 44). We controlled for sex and used focal subject individual as a random effect. In the second full model, we modeled the proportion of unimodal to multimodal signals produced within each focal observation. This allowed us to account for different observational efforts across contexts. We included signal sender age as a continuous predictor and behavioral context as a fixed effect, while controlling for sex and including observation ID nested inside of focal subject ID as a random factor.

We checked for overdispersion and convergence issues. We detected no overdispersion and found good convergence in both full models. Full models were compared to corresponding null models in which the test predictors were removed (i.e. age in the first model, age and behavioral context in the second model). This comparison was done using analysis of variance tests (ANOVA) with the “anova” function in the “stats” package (R Core Team 2023). As the full models were significantly better than the null models, we tested the significance of the single predictors using the function “drop1” with a “Chisq” test. For behavioral contexts, we ran post-hoc pairwise comparisons between all contexts using Tukey adjustments and the “emmeans” function and package (Lenth 2025).

## Results

### Overview of signal production

From the total 7,617 communication signals observed, 6,876 were unimodal signals and 741 were multimodal signals (Table 3). Among modalities, gestures were the most prevalent composing 75.63% of all unimodal signals (5,200 signals). Vocalizations were the second most used signal type occurring 1,238 times (18.00% of all unimodal signals). Finally, facial expressions were observed the least and were 6.37% of all unimodal signals (438 signals).

**Table 3:** Communication signals sent within each modality and age category

Age category	Modality	Number observed
Infant (1)	Facial expression	148
	Gesture	971
	Vocalization	13
	Multimodal	224
Juveniles (2)	Facial expression	148
	Gesture	2,294
	Vocalization	55
	Multimodal	310
Adolescents (3)	Facial expression	79
	Gesture	977
	Vocalization	288
	Multimodal	157
Adults (6)	Facial expression	63
	Gesture	958
	Vocalization	882
	Multimodal	50

**Fig. 1:**
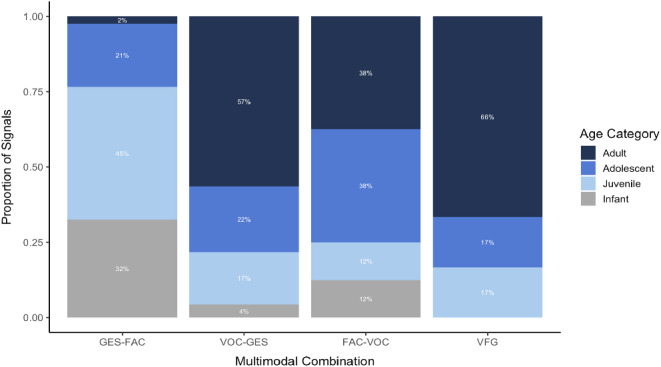
The proportion of multimodal signal combinations produced by bonobos in each age category (*n* = 12). Combinations of multimodal signals are noted as gestures + facial expressions (GES-FAC), vocalizations + gestures (VOC-GES), and facial expressions + vocalizations (FAC-VOC). All 3 modalities together are denoted as VOC + FAC + GES (not seen in the infant age category)

A total of 741 multimodal signals were observed, making them rare compared to the number of unimodal signals. Although multimodal signals were relatively rare, they were sent by all individuals in the sample. Combinations and production of multimodal signals varied by age category (Fig. 1). Multimodal signals composed of gestures + facial expressions were the most recorded combination (681 signals; adult: 17; adolescent: 143, juveniles: 300, infant: 221). Vocalizations + gestures were observed 46 total times (adult: 26, adolescents: 10, juveniles: 8, infant: 2), and facial expressions + vocalizations were only seen 8 times (adult: 3, adolescents: 3, juveniles: 1, infant: 1). Multimodal combinations across all three modalities, vocalization + facial expression + gesture, were rarely observed (6 signals; adult: 4, adolescents: 1, juveniles: 1, infant: 0).

### Proportion of signals related to age

**Fig. 2:**
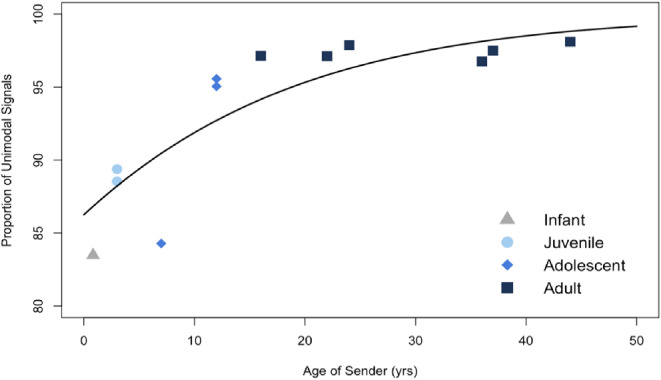
Relationship between bonobo age and the proportion of unimodal communication signals sent (*n* = 12). Each data point represents a single individual, with age plotted on the x-axis and the proportion of unimodal signals sent on the y-axis. Point shape and color indicate age category for each bonobo. The black line is the predicted unimodal signal proportions from the Model 1 GLMM

Model 1 assessed whether the relative proportion of unimodal to multimodal communication signals increased through age (Prediction 2). The comparison between our full model 1 and the corresponding null model indicated that the full model was significantly better than the null one (df = 1, 𝑋^2^ = 15.34, *p* < 0.001). Signaler age was highly significant, with younger bonobos producing more multimodal signals than older bonobos (*p* < 0.001; Table 4). The regression coefficient (β = 0.06) indicated that for each age year increase, unimodal signal production increases approximately 6% (OR = 1.06; Fig. 2). An additional figure with proportions of unimodal and multimodal signal production by age category can be found in the Supplementary Information (Fig. S2). Sex was not significant in this model (*p* = 0.432).

**Table 4:** Statistical model results with reported estimates, standard error (SE), confidence intervals (CI), an *p*-values (* denotes statistical significance)

Models	Estimate	SE	2.5–97.5% CI	*P*-value
**Model 1: Probability of multimodal signal production**				
Intercept	-1.84	0.24	-2.30 to -1.37	-
Signaler age	-0.06	0.01	-0.08 to -0.04	< 0.001*
Sex (male)	-0.21	0.26	-0.72 to 0.30	0.432
**Model 2: Proportion of unimodal to multimodal signals produced by context**				
Intercept	4.89	0.73	3.46 to 6.32	-
Signaler age	0.03	0.01	0.01 to 0.04	0.005*
Behavioral context	-4.01	0.77	-5.49 to -2.53	< 0.001*
Sex (male)	0.45	0.11	0.23 to 0.66	0.008*

### Behavioral context of multimodal signals

**Fig. 3:**
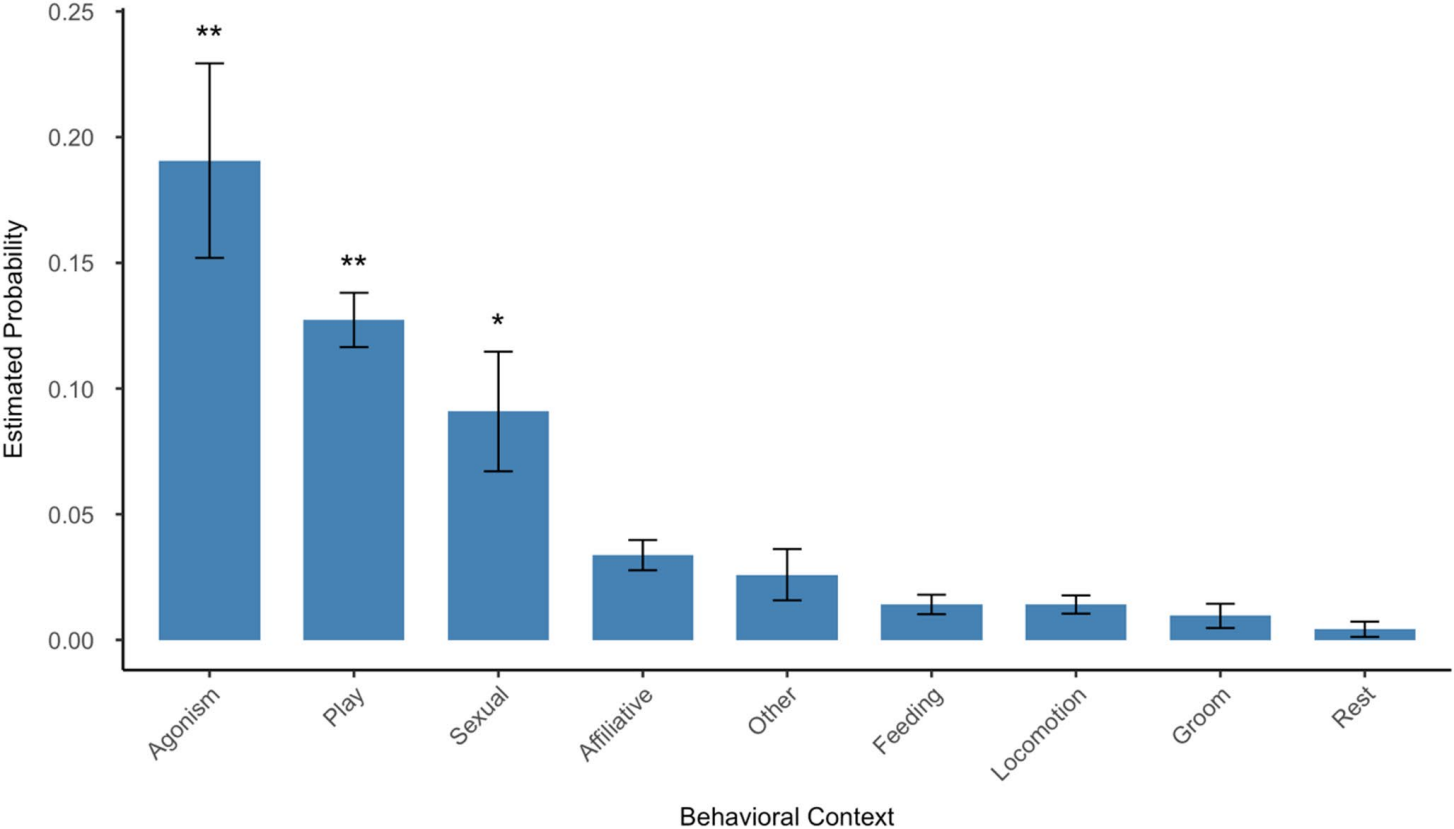
Probability of multimodal signal production in different behavioral contexts. Asterisks denote significant contexts compared to the reference rest context (* *p* < 0.01, ** *p* < 0.001)

Model 2 assessed the impact of behavioral context (Prediction 1) and age (Prediction 2) on the production of multimodal signals. The full-null model comparison showed that the full model was significantly better than the null one (df = 9, 𝑋^2^ = 340.79, *p* < 0.001). Both age (*p* = 0.005) and context (*p* < 0.001) had a significant effect on the proportion of unimodal to multimodal signals produced within each focal observation (Table 4). Post-hoc pairwise comparisons showed that the relative proportion of unimodal signals was overall significantly lower in agonistic, play, and sexual contexts as compared to other contexts (agonistic-affiliative, agonistic-feeding, agonistic-groom, agonistic-locomotion, agnostic-rest, agonistic-other: all *p* < 0.001; play-affiliative, play-feeding, play-groom, play-locomotion, play-rest: all *p* < 0.001, play-other *p* = 0.002; sexual-feeding and sexual-locomotion: *p* < 0.001, sexual-affiliative *p* = 0.045, sexual-groom *p* = 0.002, sexual-rest *p* = 0.001; Fig. 3). For the complete list of estimates and Tukey’s pairwise post-hoc comparisons, see the Supplementary Information Table S5. Moreover, the relative proportion of unimodal to multimodal signals increased with age, as seen in Model 1. A table displaying the total amount of unimodal and multimodal signals produced by each age category across the behavioral contexts can be found in the Supplementary Information (Table S6). Finally, sex was significant in this model, with males producing relatively more unimodal signals than female bonobos (*p* = 0.008).

## Discussion

Few studies have assessed if great apes produce unimodal or multimodal communication signals at different rates (Hobaiter et al. 2017; Wilke et al. 2017; Doherty et al. 2023). Previous work has assessed a few multimodal signals, one modality, or a specific multimodal combination in a certain behavioral context (Leavens et al. 2009; Genty et al. 2014; Taglialatela et al. 2015; Wilke et al. 2017; Genty 2019; Mine et al. 2024). Our results provide the first bonobo age comparison of the production of unimodal and multimodal signal combinations across all three main modalities (facial expressions, gestures, and vocalizations) and in a variety of contexts. By examining how different aged individuals utilize these signals, this study provides an overview of the developmental differences in bonobo communication patterns.

Bonobos across all ages sent more unimodal signals than multimodal signals suggesting it is their preferred mode of communication. Unimodal signals were largely gestures, which were the most frequently used signal type regardless of age and context. The infant bonobo in this study, at six months of age, displayed the highest individual percentage of multimodal signals combining facial expressions and gestural modalities. However, in terms of age category, juveniles produced the most multimodal signals. Following our Prediction 2, rates of multimodality declined into adolescence and further into adulthood. Adult bonobos sent proportionately fewer multimodal signals than any others but favored multimodal combinations of vocalization and gestures. This age trajectory followed an increase of approximately 6% more unimodal signals with each year of age. These results indicate that age is highly predictive of multimodal signal usage with the highest rates in younger bonobos (Prediction 2). Results in wild chimpanzees have also observed this trend noting that multimodal signals occur rarely and there is a trend with increasing age to switch and send more unimodal signals (Fröhlich et al. 2016). Taken together, this suggests that multimodal signaling is primarily utilized by younger individuals and may be a crucial component of learning how to effectively communicate with other bonobos.

Past research has investigated *Pan* use of multimodal signals in specific contexts such as sexual behaviors, play, and post-conflict interactions but these results are mostly limited to a singular context in each study (Genty et al. 2014; Wilke et al. 2017; Grampp et al. 2023). Here, we looked across nine behavioral contexts finding significance and increased use of multimodal signals in three contexts- agonistic, play, and sexual behaviors (Prediction 1a-b). All individuals except the infant, who was not observed in any agonistic contexts, sent multimodal signals during these types of behaviors. This finding is consistent with research in chimpanzees, where multimodal communication is often employed during or immediately following agonistic interactions (Genty et al. 2014; Hobaiter et al. 2017; Grampp et al. 2023). Play contexts were also found to be statistically associated with increased multimodality (Prediction 1b). These results of increased multimodality are similar to what Doherty et al. (2023) observed in chimpanzee play contexts, although their results were only inclusive of individuals aging from infancy to adolescence. For younger individuals specifically, it could be that they can more freely engage in and practice multimodal signals within play contexts (Cordoni and Palagi 2011; Demuru et al. 2015; Palagi et al. 2015). Sexual contexts also showed an increase in multimodal signaling, particularly in adults. Two of the statistically significant contexts, agonism and sexual, are “high-risk/high-reward” behavioral situations, which we predicted would show increased multimodal signaling (Prediction 1a). An important function of multimodal communication signals is to aid in proper receiver comprehension by clarifying the meaning of one signal with the addition of a second modality (Genty et al. 2014; Hobaiter et al. 2017; Wilke et al. 2017; Fröhlich et al. 2019). In behavioral contexts that have high risk or reward, such as aggression or sexual encounters, proper signal comprehension is vital. Combining different unimodal signals may allow signal senders to select signals that convey a clear and undeniable meaning to their receivers. By analyzing how bonobos use multimodal signals during certain activities, these results aid in a more comprehensive understanding of how context impacts signal type. Thus, age, as well as context, are predictors of which modalities are utilized in bonobo communication.

Results from this study mirror the repertoire tuning hypothesis proposed to explain serial gesturing in chimpanzees; where younger individuals produce more rapid sequences of gestures and taper off through age as individuals learn the meaning of gestures (Hobaiter and Byrne 2011a). We similarly propose that the observed decline in multimodal communication with age reflects a developmental progression toward a more streamlined repertoire of unimodal signals. By combining multiple modalities, senders have a greater chance of eliciting a satisfactory outcome from their signal receivers. For young bonobos, the ability to combine signals in various ways could offer a flexible and adaptive strategy for engaging with social partners. This suggests that as bonobos mature, they learn individual signal meanings and can then rely more on simplified unimodal signals. Recent work studying unimodal and multimodal signal production across chimpanzees from infancy to early adolescence found that multimodal communication is more prevalent in adolescent individuals than in infants and juveniles (Doherty et al. 2023). While the authors did not compare signaling rates in adult individuals, there are results in the literature that suggest in adult chimpanzees, there is no age-based effect on rates of multimodal signals (Hobaiter and Byrne 2011b; Hobaiter et al. 2017; Wilke et al. 2017). This contradicts the clear age difference presented in the current study. The present results could indicate that bonobos and chimpanzees use multimodal signals differently despite their close phylogenetic relationship.

This study provides critical insight into how age affects the use of unimodal and multimodal communication signals in bonobos. A limitation of this study is the sample size, particularly regarding the single infant. This limits the findings as a singular individual cannot represent all individuals. Additionally, this work took place in one population of captive animals which may not be representative of other populations or wild bonobo behaviors. Research on wild bonobos and other captive colonies will allow us to assess whether the findings observed here are consistent with other environments.

More work is needed to understand the meaning of multimodal communication signals, especially those utilized by bonobos. Investigating the functional roles of multimodal signal combinations could provide deeper insights into the factors driving bonobo communication. While we controlled for sex and it was not significant in our age model, it was significant in the model on behavioral contexts. Taking a closer look at the effect of sex on multimodal signal rates could be beneficial in future work. Finally, researchers should consider focusing more on traits of signal senders in future studies such as rank, social bonds, receiver species, physical proximity, and sexual receptivity to see how they influence bonobos’ use of unimodal versus multimodal communication signals.

No research had yet taken a complete view of all gestural, vocal, and facial combinations of multimodal signals in bonobos. This study contributes to the understanding of multimodal signal production in bonobos, by simultaneously assessing their use of gestural, vocal and facial combinations. By comparing individuals across ontogeny, multiple modalities and contexts, we shed light onto the development of multimodal communication in our closest living relatives. Our results also provide a new methodological approach for future communication research. These results underscore the importance of age and context on signal modality, suggesting that the use of multimodal communication is a key component of the communication-forming process in younger bonobos.

## Electronic supplementary material

Below is the link to the electronic supplementary material.

Supplementary Material 1

## Data Availability

The data that support the findings of this study are available from the corresponding author upon request and approval from the authors and Apenheul Primate Park’s zoological manager and research coordinator.

## Code availability

All code for data analysis for these results was run in R. Code associated with this study is available with written consent from the corresponding author and the zoological manager and research coordinators at Apenheul Primate Park.

## References

[CR1] Altmann J (1974) Observational study of behavior: sampling methods. Behaviour 49(3):227–266 4597405 10.1163/156853974x00534

[CR2] Amici F, Liebal K (2022) The social dynamics of complex gestural communication in great and lesser apes (Pan troglodytes, Pongo Abelii, Symphalangus syndactylus). Philosophical Trans Royal Soc B: Biol Sci 377(1860). 10.1098/rstb.2021.0299 10.1098/rstb.2021.0299 PMC9358312 35934967

[CR3] Arbib MA, Liebal K, Pika S (2008) Primate vocalization, gesture, and the evolution of human Language. Curr Anthropol 49(6):1053–1076. 10.1086/593015 19391445 10.1086/593015

[CR4] Bard KA, Dunbar S, Maguire-Herring V, Veira Y, Hayes KG, McDonald K (2014) Gestures and social-emotional communicative development in chimpanzee infants. Am J Primatol 76(1):14–29. 10.1002/ajp.22189 24038115 10.1002/ajp.22189

[CR6] Bermejo M, Omedes A (1999) Preliminary vocal repertoire and vocal communication of wild bonobos *(Pan paniscus)* at Lilungu (Democratic Republic of Congo). Folia Primatol 70(6):328–357. 10.1159/000021717 10.1159/000021717 10640882

[CR7] Birchenall LB (2016) Animal communication and human Language: an overview. Int J Comp Psychol 29(1). 10.46867/ijcp.2016.29.00.07

[CR8] Brooks ME, Kristensen K, Benthem KJV, Magnusson A, Berg CW, Nielsen A, Skaug HJ, Mächler M, Bolker BM (2017) GlmmTMB balances speed and flexibility among packages for zero-inflated generalized linear mixed modeling. R J 9(2):378. 10.32614/rj-2017-066

[CR9] Byrne RW, Cartmill E, Genty E, Graham KE, Hobaiter C, Tanner J (2017) Great ape gestures: intentional communication with a rich set of innate signals. Anim Cogn 20(4):755–769. 10.1007/s10071-017-1096-4 28502063 10.1007/s10071-017-1096-4 PMC5486474

[CR10] Cartmill EA, Byrne RW (2010) Semantics of primate gestures: intentional meanings of orangutan gestures. Anim Cogn 13(6):793–804. 10.1007/s10071-010-0328-7 20563619 10.1007/s10071-010-0328-7

[CR11] Chevalier-Skolnikoff S (1974) The primate play face: A possible key to the determinants and evolution of play. Rice University Studies

[CR12] Cohen J (1960) A coefficient of agreement for nominal scales. Educ Psychol Meas 20(1):37–46. 10.1177/001316446002000104

[CR13] Cordoni G, Palagi E (2011) Ontogenetic trajectories of chimpanzee social play: similarities with humans. PLoS ONE 6(11):e27344 22110630 10.1371/journal.pone.0027344 PMC3217932

[CR14] D’Août K, Aerts P, Berillon G (2014) Using primate models to study the evolution of human locomotion: concepts and cases. BMSAP 26(3–4):105–110. 10.1007/s13219-014-0102-5

[CR15] Dafreville M, Hobaiter C, Guidetti M, Sillam-Dussès D, Bourjade M (2021) Sensitivity to the communicative partner’s attentional State: A developmental study on mother–infant dyads in wild chimpanzees (*Pan troglodytes schweinfurthii*). Am J Primatol 83(12). 10.1002/ajp.23339 10.1002/ajp.23339 34633101

[CR16] de Waal FBM (1988) The communicative repertoire of captive bonobos (*Pan paniscus*), compared to that of chimpanzees. Behaviour 106(3–4):183–251. 10.1163/156853988x00269

[CR17] de Waal FBM (1990) Sociosexual behavior used for tension regulation in all age and sex combinations among bonobos. Springer EBooks 378–393. 10.1007/978-1-4613-9682-6_15

[CR18] Demuru E, Ferrari PF, Palagi E (2015) Emotionality and intentionality in bonobo playful communication. Anim Cogn 18(1):333–344. 10.1007/s10071-014-0804-6 25204682 10.1007/s10071-014-0804-6

[CR19] Doherty E, Davila-Ross M, Clay Z (2023) Multimodal communication development in semiwild chimpanzees. Anim Behav 201:175–190. 10.1016/j.anbehav.2023.03.020

[CR20] Fedurek P, Slocombe KE (2011) Primate vocal communication: A useful tool for Understanding human speech and Language evolution? Hum Biol 83(2):153–173. 10.3378/027.083.0202 21615284 10.3378/027.083.0202

[CR21] Fisher SE, Marcus GF (2006) The eloquent Ape: genes, brains and the evolution of Language. Nat Rev Genet 7(1):9–20. 10.1038/nrg1747 16369568 10.1038/nrg1747

[CR22] Flack JC, Jeannotte LA, de Waal FBM (2004) Play signaling and the perception of social rules by juvenile chimpanzees (*Pan troglodytes*). J Comp Psychol 118(2):149–159. 10.1037/0735-7036.118.2.149 15250802 10.1037/0735-7036.118.2.149

[CR23] Forrester G, Forrester NA (2005) Methodology for detecting multimodal communication in Western lowland gorillas. Am J Primatol 66:159–19915940707

[CR24] Friard O, Gamba M (2016) BORIS: A free, versatile open-source event-logging software for video/audio coding and live observations. Methods Ecol Evol 7(11):1325–1330. 10.1111/2041-210x.12584

[CR25] Fröhlich M, Hobaiter C (2018) The development of gestural communication in great apes. Behav Ecol Sociobiol 72(12). 10.1007/s00265-018-2619-y

[CR28] Fröhlich M, Wittig RM, Pika S (2016) Should I stay or should I go? Initiation of joint travel in mother–infant dyads of two chimpanzee communities in the wild. Anim Cogn 19(3):483–500. 10.1007/s10071-015-0948-z 26833496 10.1007/s10071-015-0948-z PMC4824811

[CR26] Fröhlich M, Wittig RM, Pika S (2018) The ontogeny of intentional communication in chimpanzees in the wild. Dev Sci 22(1):e12716. 10.1111/desc.12716 30156360 10.1111/desc.12716

[CR27] Fröhlich M, Sievers C, Townsend SW, Gruber T, Schaik CP (2019) Multimodal communication and Language origins: integrating gestures and vocalizations. Biol Rev 94(5):1809–1829. 10.1111/brv.12535 31250542 10.1111/brv.12535

[CR29] Gamer M, Fellows I, Lemon J, Singh P (2022) Package irr (Version 0.84.1) [Computer software]. https://cran.r-project.org/web/packages/irr/irr.pdf

[CR30] Genty E (2019) Vocal–gestural combinations in infant bonobos: new insights into signal functional specificity. Anim Cogn 22(4):505–518. 10.1007/s10071-019-01267-0 31098849 10.1007/s10071-019-01267-0

[CR31] Genty E, Clay Z, Hobaiter C, Zuberbühler K (2014) Multi-modal use of a socially directed call in bonobos. PLoS ONE 9(1):e84738. 10.1371/journal.pone.0084738 24454745 10.1371/journal.pone.0084738 PMC3893130

[CR32] Gillespie-Lynch K, Greenfield PM, Lyn H, Savage-Rumbaugh S (2014) Gestural and symbolic development among apes and humans: Support for a multimodal theory of language evolution. Front Psychol 5. 10.3389/fpsyg.2014.01228 10.3389/fpsyg.2014.01228 PMC4214247 25400607

[CR33] Graham KE, Furuichi T, Byrne RW (2016) The gestural repertoire of the wild bonobo (*Pan paniscus*): a mutually understood communication system. Anim Cogn 20(2):171–177. 10.1007/s10071-016-1035-9 27632158 10.1007/s10071-016-1035-9 PMC5306194

[CR34] Grampp M, Samuni L, Girard-Buttoz C, León J, Zuberbühler K, Tkaczynski P, Wittig RM, Crockford C (2023) Social uncertainty promotes signal complexity during approaches in wild chimpanzees (*Pan troglodytes verus*) and mangabeys (*Cercocebus atys atys*). Royal Soc Open Sci 10(11). 10.1098/rsos.231073 10.1098/rsos.231073 PMC10685125 38034119

[CR35] Hashimoto C (1997) Context and development of sexual behavior of wild bonobos (*Pan paniscus*) at Wamba, Zaire. Int J Primatol 18(1):1–21. 10.1023/a:1026384922066

[CR36] Higham JP, Hebets EA (2013) An introduction to multimodal communication. Behav Ecol Sociobiol 67(9):1381–1388. 10.1007/s00265-013-1590-x

[CR37] Hobaiter C, Byrne RW (2011a) Serial gesturing by wild chimpanzees: its nature and function for communication. Anim Cogn 14(6):827–838. 10.1007/s10071-011-0416-3 21562816 10.1007/s10071-011-0416-3

[CR38] Hobaiter C, Byrne RW (2011b) The gestural repertoire of the wild chimpanzee. Anim Cogn 14(5):745–767. 10.1007/s10071-011-0409-2 21533821 10.1007/s10071-011-0409-2

[CR39] Hobaiter C, Byrne RW, Zuberbühler K (2017) Wild chimpanzees’ use of single and combined vocal and gestural signals. Behav Ecol Sociobiol 71(6). 10.1007/s00265-017-2325-1 10.1007/s00265-017-2325-1 PMC5446553 28596637

[CR42] Jensen-Seaman MI, Deinard AS, Kidd KK (2001) Modern African ape populations as genetic and demographic models of the last common ancestor of humans, chimpanzees, and gorillas. J Hered 92(6):475–480. 10.1093/jhered/92.6.475 11948214 10.1093/jhered/92.6.475

[CR43] Kuhl PK (2010) Brain mechanisms in early Language acquisition. Neuron 67(5):713–727. 10.1016/j.neuron.2010.08.038 20826304 10.1016/j.neuron.2010.08.038 PMC2947444

[CR45] Leavens DA (2007) Animal cognition: multimodal tactics of orangutan communication. Curr Biol 17(17):R762–R764. 10.1016/j.cub.2007.07.010 17803926 10.1016/j.cub.2007.07.010

[CR46] Leavens DA, Hostetter AB, Wesley MJ, Hopkins WD (2004) Tactical use of unimodal and bimodal communication by chimpanzees, *Pan troglodytes*. Anim Behav 67(3):467–476. 10.1016/j.anbehav.2003.04.007

[CR47] Leavens DA, Russell JL, Hopkins WD (2009) Multimodal communication by captive chimpanzees (*Pan troglodytes*). Anim Cogn 13(1):33–40. 10.1007/s10071-009-0242-z 19504272 10.1007/s10071-009-0242-z PMC2797826

[CR48] Lenth RV (2025) Package emmeans: estimated marginal means, aka Least-Squares means (Version 1.11.0–005) [Computer software]. https://rvlenth.githun.io/emmeans/

[CR49] Levinson SC, Holler J (2014) The origin of human multi-modal communication. Philosophical Trans Royal Soc B: Biol Sci 369(1651):20130302–20130302. 10.1098/rstb.2013.0302 10.1098/rstb.2013.0302 PMC4123681 25092670

[CR50] Luke S, Verma RS (1995) Human (*Homo sapiens*) and chimpanzee (*Pan troglodytes*) share similar ancestral centromeric alpha satellite DNA sequences, but other fractions of heterochromatin differ considerably. Am J Phys Anthropol 96(1):63–71. 10.1002/ajpa.1330960107 7726296 10.1002/ajpa.1330960107

[CR51] Manson JH, Perry S, Parish AR (1997) Nonconceptive sexual behavior in bonobos and capuchins. Int J Primatol 18(5):767–786. 10.1023/A:1026395829818

[CR53] Mayberry RI, Lock E, Kazmi H (2002) Linguistic ability and early Language exposure. Nature 417(6884):38–38. 10.1038/417038a 11986658 10.1038/417038a

[CR54] Mine JG, Wilke C, Zulberti C, Behjati M, Bosshard AB, Stoll S, Machanda ZP, Manser A, Slocombe KE, Townsend SW (2024) Vocal-visual combinations in wild chimpanzees. Behav Ecol Sociobiol 78(10). 10.1007/s00265-024-03523-x

[CR55] Oliveira MFDS, Wasterlain SN (2020) How zoo-housed chimpanzees (*Pan troglodytes*) target gestural communication within and between age groups. Antropologia Portuguesa 37:7–28. 10.14195/2182-7982_37_1

[CR56] Oña LS, Sandler W, Liebal K (2019) A stepping stone to compositionality in chimpanzee communication. PeerJ 7:e7623. 10.7717/peerj.7623 10.7717/peerj.7623 PMC6745191 31565566

[CR57] Palagi E (2006) Social play in bonobos (*Pan paniscus*) and chimpanzees (*Pan troglodytes*): implications for natural social systems and interindividual relationships. Am J Phys Anthropol 129(3):418–426. 10.1002/ajpa.20289 16323189 10.1002/ajpa.20289

[CR58] Palagi E, Burghardt GM, Smuts B, Cordoni G, Dall’Olio S, Fouts HN, Řeháková-Petrů M, Siviy SM, Pellis SM (2015) Rough‐and‐tumble play as a window on animal communication. Biol Rev 91(2):311–327. 10.1111/brv.12172 25619897 10.1111/brv.12172

[CR59] Parish AR, De Waal FBM, Haig D (2000) The other closest living relative: how bonobos (*Pan paniscus*) challenge traditional assumptions about females, dominance, intra- and intersexual interactions, and hominid evolution. Ann N Y Acad Sci 907(1):97–113. 10.1111/j.1749-6632.2000.tb06618.x 10818623

[CR60] Parr LA (2004) Perceptual biases for multimodal cues in chimpanzee (*Pan troglodytes*) affect recognition. Anim Cogn 7(3). 10.1007/s10071-004-0207-1 10.1007/s10071-004-0207-1 14997361

[CR61] Partan S, Marlar P (1999) Communication goes multimodal. Science 283(5406):1272–1273. 10.1126/science.283.5406.1272 10084931 10.1126/science.283.5406.1272

[CR62] Partan SR, Marler P (2005) Issues in the classification of multimodal communication signals. Am Nat 166(2):231–245. 10.1086/431246 16032576 10.1086/431246

[CR63] Pika S, Liebal K, Call J, Tomasello M (2005) The gestural communication of apes. In: Liebal K, Müller C, Pika S (eds) Gestural communication in nonhuman and human primates. Benjamin’s current topics, vol 10. John Benjamins Publishing Company, Amsterdam, pp 35–49

[CR64] Pollick AS, de Waal FBM (2007) Ape gestures and Language evolution. Proc Natl Acad Sci 104(19):8184–8189. 10.1073/pnas.0702624104 17470779 10.1073/pnas.0702624104 PMC1876592

[CR65] Prüfer K, Munch K, Hellmann I, Akagi K, Miller JR, Walenz B, Koren S, Sutton G, Kodira C, Winer R, Knight JR, Mullikin JC, Meader SJ, Ponting CP, Lunter G, Higashino S, Hobolth A, Dutheil J, Karakoç E, Alkan C (2012) The bonobo genome compared with the chimpanzee and human genomes. Nature 486(7404):527–531. 10.1038/nature11128 22722832 10.1038/nature11128 PMC3498939

[CR66] R Core Team (2023) R: A language and environment for statistical computing [Computer software]. R Foundation for Statistical Computing, Vienna, Austria. https://www.R-project.org/

[CR67] Rodrigues ED, Fröhlich M (2021) Operationalizing intentionality in primate communication: social and ecological considerations. Int J Primatol 45. 10.1007/s10764-021-00248-w

[CR68] Ross M, Niemann T, Wark J, Heintz M, Horrigan A, Cronin K, Shender M, Gillespie K (2016) ZooMonitor (Version 1) [Computer software]. https://zoomonitor.org

[CR69] Sayers K, Lovejoy CO (2008) The chimpanzee has no clothes: A critical examination of *Pan troglodytes* in models of human evolution [with comments]. Curr Anthropol 49(1):87. 10.2307/20142605

[CR70] Schneider C, Call J, Liebal K (2011) What role do mothers play in the gestural acquisition of bonobos (*Pan paniscus*) and chimpanzees (*Pan troglodytes*)? Int J Primatol 33(1):246–262. 10.1007/s10764-011-9570-3

[CR71] Schneider C, Liebal K, Call J (2017) Giving and responding differences in gestural communication between nonhuman great ape mothers and infants. Dev Psychobiol 59(3):303–313. 10.1002/dev.21495 28323346 10.1002/dev.21495 PMC5434908

[CR72] Seyfarth RM, Cheney DL, Harcourt AH, Stewart KJ (1994) The acoustic features of gorilla double grunts and their relation to behavior. Am J Primatol 33(1):31–50. 10.1002/ajp.1350330104 31936924 10.1002/ajp.1350330104

[CR73] Slocombe KE, Waller BM, Liebal K (2011) The Language void: the need for multimodality in primate communication research. Anim Behav 81(5):919–924. 10.1016/j.anbehav.2011.02.002

[CR75] Taglialatela JP, Russell JL, Schaeffer JA, Hopkins WD (2008) Communicative signaling activates Broca’s homolog in chimpanzees. Curr Biol 18(5):343–348. 10.1016/j.cub.2008.01.049 18308569 10.1016/j.cub.2008.01.049 PMC2665181

[CR76] Taglialatela JP, Russell JL, Schaeffer JA, Hopkins WD (2011) Chimpanzee vocal signaling points to a multimodal origin of human Language. PLoS ONE 6(4):e18852. 10.1371/journal.pone.0018852 21533079 10.1371/journal.pone.0018852 PMC3080370

[CR74] Taglialatela JP, Russell JL, Pope SM, Morton T, Bogart S, Reamer LA, Schapiro SJ, Hopkins WD (2015) Multimodal communication in chimpanzees. Am J Primatol 77(11):1143–1148. 10.1002/ajp.22449 26212686 10.1002/ajp.22449 PMC5038593

[CR77] Tanner JE, Perlman M (2017) Moving beyond ‘meaning’: gorillas combine gestures into sequences for creative display. Lang Commun 54:56–72. 10.1016/j.langcom.2016.10.006

[CR78] Tomasello M (2008) Origins of human communication, 1st edn. MIT Press

[CR79] Tsao F-M, Liu H-M, Kuhl PK (2004) Speech perception in infancy predicts Language development in the second year of life: A longitudinal study. Child Dev 75(4):1067–1084. 10.1111/j.1467-8624.2004.00726.x 15260865 10.1111/j.1467-8624.2004.00726.x

[CR80] Vigliocco G, Perniss P, Vinson D (2014) Language as a multimodal phenomenon: implications for Language learning, processing and evolution. Philosophical Trans Royal Soc B: Biol Sci 369(1651):20130292. 10.1098/rstb.2013.0292 10.1098/rstb.2013.0292 PMC4123671 25092660

[CR82] Waller BM, Dunbar RIM (2005) Differential behavioural effects of silent bared teeth display and relaxed open mouth display in chimpanzees (*Pan troglodytes*). Ethology 111(2):129–142. 10.1111/j.1439-0310.2004.01045.x

[CR81] Waller BM, Caeiro CC, Davila-Ross M (2015) Orangutans modify facial displays depending on recipient attention. PeerJ 3:e827. 10.7717/peerj.827 25802802 10.7717/peerj.827 PMC4369341

[CR83] Weisberg DS, Zosh JM, Hirsh-Pasek K, Golinkoff RM (2013) Talking it up: play, Language development, and the role of adult support. Am J Play 6:39–54

[CR84] Wilke C, Kavanagh E, Donnellan E, Waller BM, Machanda ZP, Slocombe KE (2017) Production of and responses to unimodal and multimodal signals in wild chimpanzees, *Pan troglodytes schweinfurthii*. Anim Behav 123:305–316. 10.1016/j.anbehav.2016.10.024

[CR85] Wrangham RW (1993) The evolution of sexuality in chimpanzees and bonobos. Hum Nat 4(1):47–79 24214293 10.1007/BF02734089

