# Supplementary material for: Age differences in bonobo (Pan paniscus) multimodal communication signals
Source: Anim Cogn. 2025 May 19;28(1):38. doi: 10.1007/s10071-025-01961-2 (PMC12089177; doi:10.1007/s10071-025-01961-2)
Supplement: Supplementary file 1 — Supplementary Material 1 [file 10071_2025_1961_MOESM1_ESM.docx]

**Supplementary Information**


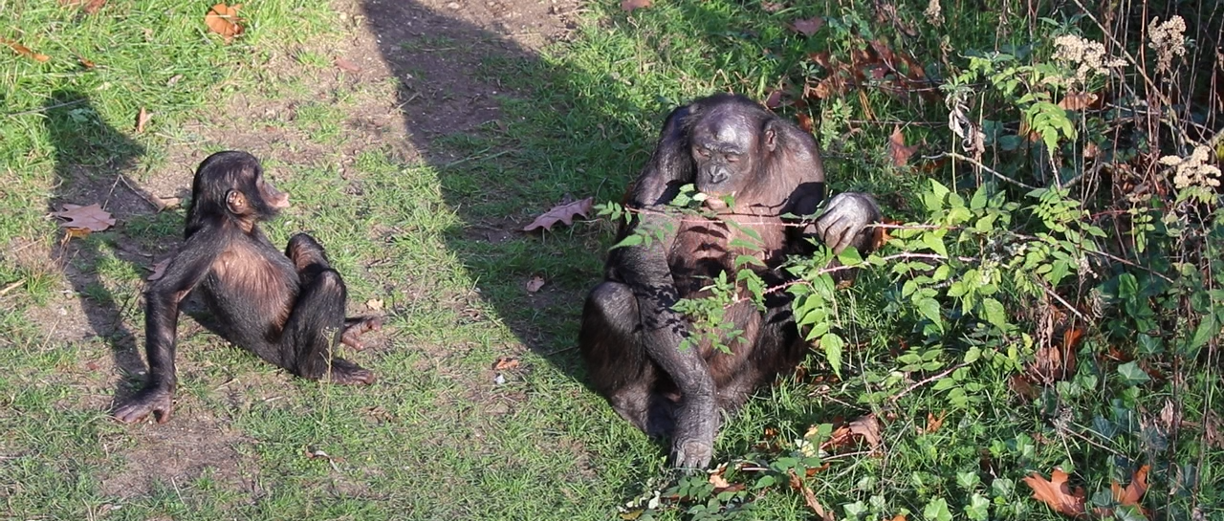


**Fig. S1** Example of a multimodal gesture + facial expression signal produced by Eyenga (left juvenile male, age 3 years). Multimodal signals are two or more communicative modalities (gestures, vocalizations, facial expressions) produced at the same time. Here, Eyenga is producing a rocking “sway” gesture by leaning back on his hands while giving a “kiss face” facial expression.


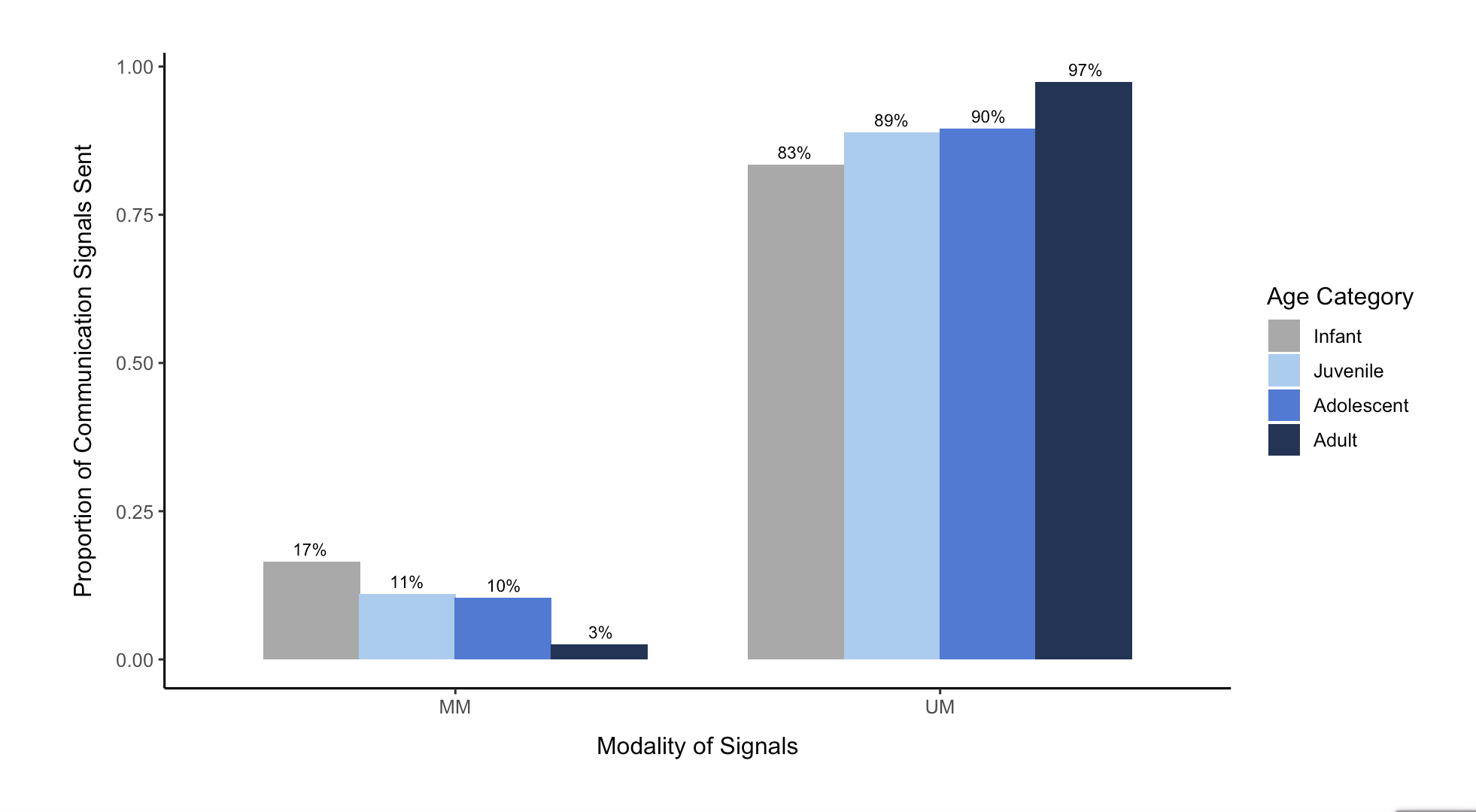


**Fig. S2** The proportion of unimodal (UM) and multimodal (MM) signals produced by bonobos in each age category (*n* = 12). The proportion of communication signals is shown along the y-axis. Age categories of signal senders, adult or non-adults, are represented by the differing colors.

**Table S1.** Ethogram of facial expressions and description of each included in this study. Facial expressions in primates are defined as voluntary facial muscular movements produced and directed towards a receiver (Burrows et al. 2006; Waller and Micheletta 2013).

**FACIAL EXPRESSIONS**

| **Code** | **Facial Expression** | **Description** |
| --- | --- | --- |
| **GM** | Grimace | Teeth exposed in a forced smile; usually produced in submission or when threatened. |
| **KF** | Kiss Face | Lips pursed and pushed forward or lips tight with chin angled upward. |
| **PF** | Play Face | Teeth partially or fully exposed, mouth relaxed in grin, often produced during a play bout. |
| **RF** | Raspberry Face | Trapping air in puffed and closed lips; may be followed by raspberry blow. |
| **FAC** | Other Facial | Distinct facial expression not meeting the above categories. |

**Table S2.** Ethogram of gestures and description of each included in this study. Gestures are considered socially directed body movements towards another individual (Hobaiter and Byrne 2011; Byrne et al. 2017; Fröhlich and Hobaiter, 2018).

**GESTURES**

| **Code** | **Gesture** | **Description** |
| --- | --- | --- |
| **AR** | Arm Raise | Upward movement of arm with palm vertical or facing downward. |
| **BA** | Bang | Hit an object with hand or wrist in a swift forceful manner. |
| **BI** | Bite | Clamping of mouth around portion of another individual; may be playful or agonistic. |
| **BW** | Bow | Lean down with head lowest in position, crouched, with bottom up in air. |
| **CL** | Clap | Slapping hands or palms together. |
| **DP** | Drag or Pull | Displacement of object or individual in a pulling or dragging manner with hands. |
| **EM** | Embrace | Placing or wrapping arms around another individual in a tender manner. |
| **FR** | Foot Raise | Lifting foot or leg off ground to an elevated manner. |
| **GI** | Genital Inspect | Peering, touching, or smelling genitals of another individual or self. |
| **GP** | Genital Present | Positioning and presenting genital regions toward another individual. |
| **GB** | Grab | Reach and grasping contact with an object or individual. |
| **HS** | Head Shake | Horizontal or vertical nodding of head; can occur in sequence or singularly. |
| **HB** | Headbutt | Hitting head against another individual. |
| **HI** | Hit | Forceful strike with hand or foot. |
| **KI** | Kick | Forceful strike with foot. |
| **OP** | Open Palm | Palm opened upward in a cupped manner. |
| **PA** | Pat | Gentle touching with hand or foot in sequence. |
| **PI** | Pirouette | Spinning in a circle on all four limbs or elevated on limbs. |
| **PJ** | Play Jump | Bouncing movement; Usually occurs to indicate the start or desire for a play bout. |
| **PT** | Point | Raised hand with 1-3 finders aligned in a distinct indicational manner. |
| **PO** | Poke | Contact with flattened and straightened fingers of hand; can occur in sequence. |
| **PC** | Pounce | Playful throwing of body onto another object or individual. |
| **PU** | Push | Forceful displacement with hands. |
| **RK** | Rap Knuckles | Hitting of knuckles onto a substrate. |
| **RE** | Reach | Attempting to grab or move arm towards an object or individual. |
| **SH** | Self-Hit | Slapping own body with hand. |
| **SO** | Somersault | Flipping movement of whole body. |
| **ST** | Stomp | Use of leg and foot to strike ground. |
| **SW** | Swagger | Movement of body side-to-side while standing bipedally; usually preceding displays. |
| **SY** | Sway | Rocking movement in quadrupedal or sitting position. |
| **SA** | Swat | Rapid reaching movement towards an individual. |
| **TH** | Throw | Using hand to toss an object. |
| **TO** | Touch | Gentle contact with hand or foot. |
| **GES** | Other Gesture | Distinct gesture not meeting the above categories. |

**Table S3.** Ethogram of vocalizations and description of each included in this study. Vocalizations are distinct tones or utterances created by the lungs, larynx, and other sound-producing organs (Jürgens 1988; Fitch and Hausser 1995).

**VOCALIZATIONS**

| **Code** | **Vocal Name** | **Description** |
| --- | --- | --- |
| **AL** | Alarm Call | Loud, sharp vocals produced in the context of real or perceived danger. Sounds like “waa” or “wra”. |
| **BR** | Bark | Sharp but guttural vocalization. |
| **CO** | Copulation Scream | Higher-pitched screams or squeals produced when copulating, pseudo-copulation, or genito-genital rubbing. |
| **GT** | Grunt | Low-pitch vocalizations usually produced under breath. |
| **HO** | Hoot | Relatively loud voiced inhales and exhales; sounds like “ooo”. |
| **LA** | Laugh | Rapid inhale and releases of air that resemble a “chuckle”. |
| **PE** | Peep | Short staccato medium or high chirp sounds; may be produced in series; often expressed during excitement. |
| **PM** | Pout-moan | Low-pitched melodious call in a two-part sequence. |
| **RB** | Raspberry Blow | Blowing out air through closed lips to produce “wheezing” sound. |
| **SC** | Scream | Loud and high-pitched vocalizations that tend to be long in tone. |
| **VOC** | Other Vocal | Distinct vocalization not meeting the above categories. |

**Table S4** Description of behavioral contexts

| **Behavioral Context** | **Description** |
| --- | --- |
| Affiliative | Positive or non-agonistic social interactions such as body contact, embraces, gentle touches, approaches, and sharing food. |
| Agonistic | Conflict or aggressive behaviors such as threats, chasing, biting, hitting, and fights. |
| Feeding | Ingestion of food items or active searching for a food item. |
| Grooming | Cleaning behavior on oneself or another individual. |
| Locomotion | Moving of body from one location to another. |
| Other | Coprophagy, regurgitation & reingestion, urinate, defecate, caretaker interactions, training sessions, etc. |
| Play | Individual or multiple individuals engaging in non-agonistic, relaxed play behaviors such as tickling, wrestling, and tumbling. |
| Rest | Little to no body movement with eyes open or closed. |
| Sexual | Socio-sexual or copulatory behavior including mounts, genito-genital rubbing, intromission, penis fencing, masturbation, genital presentation, and genital inspection. |

**Table S5** Tukey’s post-hoc pairwise comparisons of behavioral contexts with back-transformed odds of multimodal signal production. Asterisks denote significance (* ≤ 0.05, ** ≤ 0.001).

| **Pairwise Contexts** | **Odds Ratio** | **SE** | **Z** | **P-value** |
| --- | --- | --- | --- | --- |
| Rest – Agonism | 0.02 | 0.01 | -5.31 | <0.001** |
| Rest – Affiliative | 0.12 | 0.09 | -2.84 | 0.103 |
| Rest – Feeding | 0.30 | 0.23 | -1.58 | 0.816 |
| Rest – Groom | 0.44 | 0.39 | -0.93 | 0.991 |
| Rest – Locomotion | 0.30 | 0.23 | -1.59 | 0.811 |
| Rest – Other | 0.16 | 0.13 | -2.24 | 0.379 |
| Rest – Play | 0.03 | 0.02 | -4.88 | <0.001** |
| Rest – Sexual | 0.04 | 0.03 | -4.09 | 0.001** |
| Agonism – Affiliative | 6.75 | 2.11 | 6.10 | <0.001** |
| Agonism – Feeding | 16.45 | 6.03 | 7.63 | <0.001** |
| Agonism – Groom | 24.35 | 13.79 | 5.66 | <0.001** |
| Agonism – Locomotion | 16.46 | 5.91 | 7.80 | <0.001** |
| Agonism – Other | 8.84 | 4.16 | 4.62 | <0.001** |
| Agonism – Play | 1.12 | 0.45 | 1.74 | 0.722 |
| Agonism – Sexual | 2.36 | 0.89 | 2.27 | 0.363 |
| Affiliative – Feeding | 2.44 | 0.81 | 2.69 | 0.152 |
| Affiliative – Groom | 3.61 | 1.94 | 2.39 | 0.290 |
| Affiliative – Locomotion | 2.44 | 0.75 | 2.89 | 0.092 |
| Affiliative – Other | 1.31 | 0.58 | 0.61 | 1.00 |
| Affiliative – Play | 0.24 | 0.04 | -7.88 | <0.001** |
| Affiliative – Sexual | 0.35 | 0.12 | -3.14 | 0.045* |
| Feeding – Groom | 1.48 | 0.85 | 0.68 | 0.999 |
| Feeding – Locomotion | 1.00 | 0.38 | 0.00 | 1.00 |
| Feeding – Other | 0.54 | 0.26 | -1.28 | 0.94 |
| Feeding – Play | 0.10 | 0.03 | -7.83 | <0.001** |
| Feeding – Sexual | 0.14 | 0.06 | -4.91 | <0.001** |
| Groom – Locomotion | 0.68 | 0.38 | -0.69 | 0.999 |
| Groom – Other | 0.36 | 0.23 | -1.57 | 0.821 |
| Groom – Play | 0.07 | 0.03 | -5.26 | <0.001** |
| Groom – Sexual | 0.10 | 0.06 | -4.02 | 0.002* |
| Locomotion – Other | 0.54 | 0.26 | -1.31 | 0.930 |
| Locomotion – Play | 0.10 | 0.03 | -8.67 | <0.001** |
| Locomotion – Sexual | 0.14 | 0.05 | -5.08 | <0.001** |
| Other – Play | 0.18 | 0.08 | -4.07 | 0.002* |
| Other – Sexual | 0.27 | 0.13 | -2.69 | 0.150 |
| Play – Sexual | 1.46 | 0.44 | 1.27 | 0.941 |

**Table S6** Number of signals sent in each behavioral context by age category

| **Age category** | **Behavioral Context** | **Modality** | **Number observed** |
| --- | --- | --- | --- |
| Infant (1) | Affiliative | Unimodal | 109 |
|  |  | Multimodal | 8 |
|  | Agonism | Unimodal | 0 |
|  |  | Multimodal | 0 |
|  | Feeding | Unimodal | 73 |
|  |  | Multimodal | 2 |
|  | Groom | Unimodal | 14 |
|  |  | Multimodal | 1 |
|  | Locomotion | Unimodal | 161 |
|  |  | Multimodal | 3 |
|  | Other | Unimodal | 16 |
|  |  | Multimodal | 0 |
|  | Play | Unimodal | 640 |
|  |  | Multimodal | 210 |
|  | Rest | Unimodal | 28 |
|  |  | Multimodal | 0 |
|  | Sexual | Unimodal | 9 |
|  |  | Multimodal | 0 |
| Juveniles (2) | Affiliative | Unimodal | 330 |
|  |  | Multimodal | 25 |
|  | Agonism | Unimodal | 5 |
|  |  | Multimodal | 3 |
|  | Feeding | Unimodal | 136 |
|  |  | Multimodal | 2 |
|  | Groom | Unimodal | 75 |
|  |  | Multimodal | 1 |
|  | Locomotion | Unimodal | 145 |
|  |  | Multimodal | 5 |
|  | Other | Unimodal | 30 |
|  |  | Multimodal | 3 |
|  | Play | Unimodal | 1,690 |
|  |  | Multimodal | 268 |
|  | Rest | Unimodal | 39 |
|  |  | Multimodal | 1 |
|  | Sexual | Unimodal | 47 |
|  |  | Multimodal | 2 |
| Adolescents (3) | Affiliative | Unimodal | 109 |
|  |  | Multimodal | 1 |
|  | Agonism | Unimodal | 32 |
|  |  | Multimodal | 9 |
|  | Feeding | Unimodal | 146 |
|  |  | Multimodal | 4 |
|  | Groom | Unimodal | 106 |
|  |  | Multimodal | 2 |
|  | Locomotion | Unimodal | 177 |
|  |  | Multimodal | 1 |
|  | Other | Unimodal | 27 |
|  |  | Multimodal | 0 |
|  | Play | Unimodal | 623 |
|  |  | Multimodal | 137 |
|  | Rest | Unimodal | 91 |
|  |  | Multimodal | 1 |
|  | Sexual | Unimodal | 33 |
|  |  | Multimodal | 2 |
| Adults (6) | Affiliative | Unimodal | 159 |
|  |  | Multimodal | 4 |
|  | Agonism | Unimodal | 72 |
|  |  | Multimodal | 12 |
|  | Feeding | Unimodal | 585 |
|  |  | Multimodal | 6 |
|  | Groom | Unimodal | 178 |
|  |  | Multimodal | 0 |
|  | Locomotion | Unimodal | 376 |
|  |  | Multimodal | 7 |
|  | Other | Unimodal | 154 |
|  |  | Multimodal | 4 |
|  | Play | Unimodal | 83 |
|  |  | Multimodal | 6 |
|  | Rest | Unimodal | 258 |
|  |  | Multimodal | 0 |
|  | Sexual | Unimodal | 38 |
|  |  | Multimodal | 11 |

**References for Supplementary Information**

Burrows, A. M., Waller, B. M., Parr, L. A., & Bonar, C. J. (2006). Muscles of facial expression in the chimpanzee (*Pan troglodytes*): Descriptive, comparative and phylogenetic contexts. *Journal of Anatomy*, *208*(2), 153–167.<https://doi.org/10.1111/j.1469-7580.2006.00523.x>

Byrne, R. W., Cartmill, E., Genty, E., Graham, K. E., Hobaiter, C., & Tanner, J. (2017). Great ape gestures: Intentional communication with a rich set of innate signals. *Animal Cognition*, *20*(4), 755–769.<https://doi.org/10.1007/s10071-017-1096-4>

Fitch, W. T., & Hauser, M. D. (1995). Vocal production in nonhuman primates: Acoustics, physiology, and functional constraints on “honest” advertisement. *American Journal of Primatology*, *37*(3), 191–219.<https://doi.org/10.1002/ajp.1350370303>

Fröhlich, M., & Hobaiter, C. (2018). The development of gestural communication in great apes. *Behavioral Ecology and Sociobiology*, *72*(12).<https://doi.org/10.1007/s00265-018-2619-y>

Hobaiter, C., & Byrne, R. W. (2011). The gestural repertoire of the wild chimpanzee. *Animal Cognition*, *14*(5), 745–767.<https://doi.org/10.1007/s10071-011-0409-2>

Jürgens, U. (1988). Primate communication: Signaling, vocalization. *Birkhäuser Boston EBooks*, 110–112.<https://doi.org/10.1007/978-1-4899-6776-3_44>

Waller, B. M., & Micheletta, J. (2013). Facial expression in nonhuman animals. *Emotion Review*, *5*(1), 54–59.<https://doi.org/10.1177/1754073912451503>
